# Supplementary material for: Utilization of In Vivo Imaging System to Study Staphylococcal Sepsis and Septic Arthritis Progression in Mouse Model
Source: Pathogens. 2024 Aug 2;13(8):652. doi: 10.3390/pathogens13080652 (PMC11357683; doi:10.3390/pathogens13080652)
Supplement: Supplementary file 1 [file pathogens-13-00652-s001.zip › pathogens-3104712-supplementary.pdf]

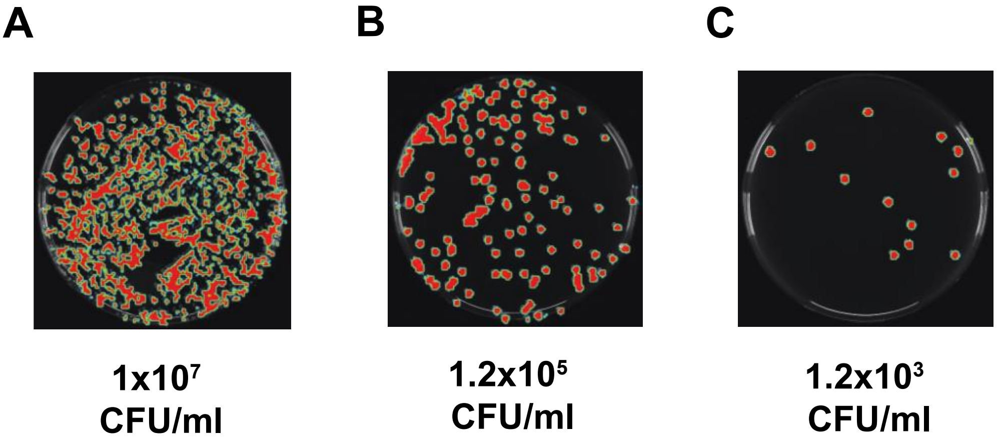

Figure S1. Bioluminescent signals from *S. aureus* AH5016 strain *in vitro*. Representative images for bioluminescent signals from serial bacterial dilutions of *S. aureus* AH5016 employed to infect mice models during the experiment. (A) higher concentration ($1x10^7$CFU/ml), (B) intermediate concentration ($1.2x10^5$ CFU/ml, and (C) lower concentration ($1.2x10^3$ CFU/ml).

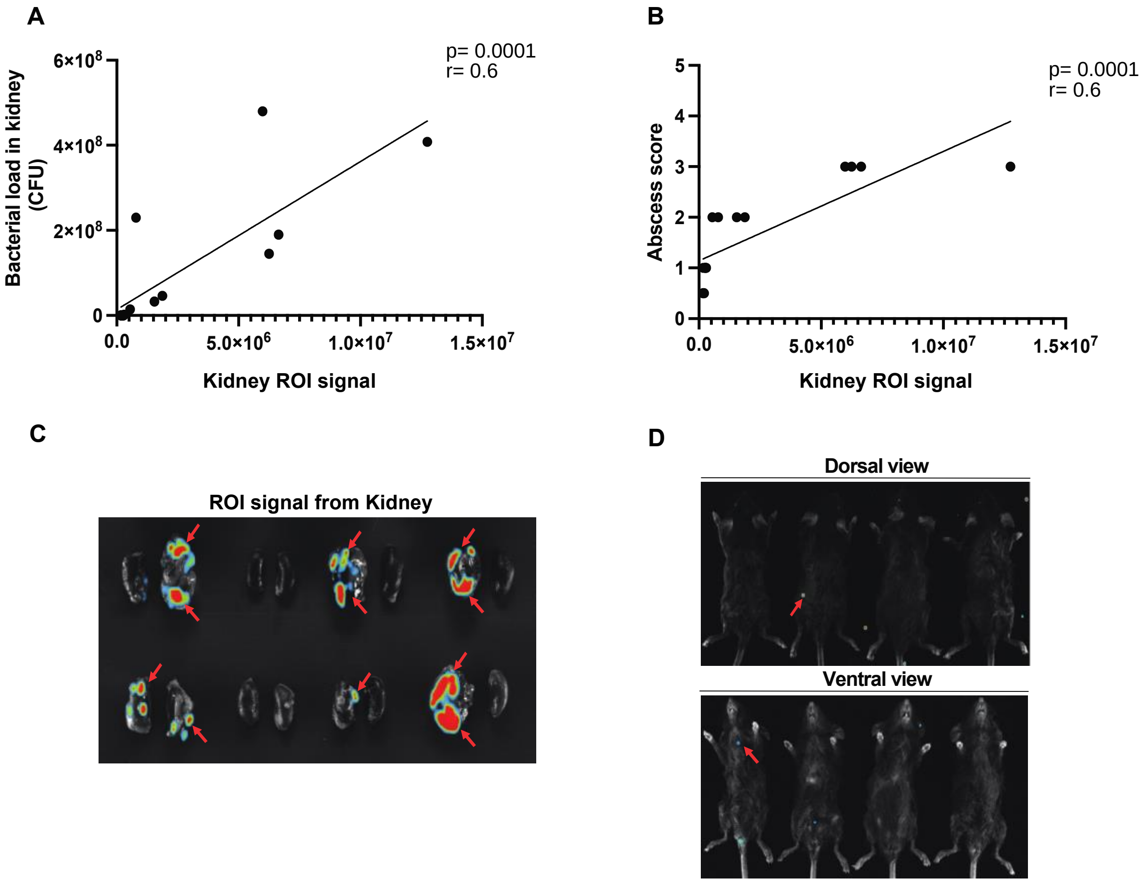

Figure S2. Kidney and whole-body imaging for assessing the severity of sepsis in C57BL/6 mice using the Newton imaging system. Correlations were studied in C57BL/6 mice (n=10) infected intravenously (i.v.) with *S. aureus* AH5016 strain (8 × 10^5 CFU/mouse). **(A)** Signals captured from kidneys in terms of region of interest (ROI) were correlated with the bacterial load in the kidneys, and **(B)** kidney abscess, **(C)** representative images of ex-vivo kidneys emitting signals and **(D)** representative images of whole mice body emitting signals. Arrows indicate bioluminescent signalling Statistical analyses were performed using the Simple linear regression and data presented as p-value and r.
